# Pigmentation and the Diagnosis of Addison's Disease

**Published:** 1908-10-10

**Authors:** Byrom Bramwell

**Affiliations:** 23 Drumsheugh Gardens, Edinburgh


### Editor's Letter-Box.

**Our correspondents are reminded that prolixity is a great bar to publication, and that brevity of style and conciseness of statement greatly facilitate early insertion.**

### PIGMENTATION AND THE DIAGNOSIS OF ADDISON'S DISEASE.

*To the Editor of* The Hospital.

Dear Sir,—In the article on "Pigmentation and the Diagnosis of Addison's Disease" in The Hospital of September 19, you state that pigmentation of the buccal mucous membrane has occasionally been observed in cases in which arsenic has been given over long periods. I am much interested in this question and will be greatly obliged if you can give me any references proving this fact. I looked up the subject some years ago and consulted, amongst others, Professor Dixon Mann, of Manchester, who has had a large experience in cases of arsenical poisoning, with the result that I could not satisfy myself that pigmentation of the buccal mucous membrane was ever produced by arsenic. It is an important question for diagnosis.

Yours very truly,
Byrom Bramwell.

23 Drumsheugh Gardens, Edinburgh.

[The question of whether pigmentation of the buccal mucosa could be a consequence of the administration of arsenic over a long period was discussed at one of the meetings of the Association of Physicians of Great Britain and Ireland, held in London in 1907, *à propos* of a remarkable case recorded verbally by Dr. Hale White. In this case the diagnosis during life lay between pernicious anæmia and Addison's disease. Dr. Hale White diagnosed the former notwithstanding the development of buccal pigmentation during the administration of arsenic, and the diagnosis of pernicious anæmia was ultimately confirmed at autopsy, the suprarenal glands not being microscopically abnormal. Whether Dr. Hale White has published this case otherwise than verbally we do not know, but we feel sure that he would willingly answer Dr. Byrom Bramwell upon this point personally.—Ed., Hospital.]

